# Evaluation of POSSUM scoring system in patients with gastric cancer undergoing D2-gastrectomy

**DOI:** 10.1186/1471-2482-5-8

**Published:** 2005-04-15

**Authors:** Elfriede Bollschweiler, Thomas Lubke, Stefan P Monig, Arnulf H Holscher

**Affiliations:** 1 Department of Visceral- and Vascular Surgery University of Cologne, Joseph-Stelzmann Str. 9, 50931 Köln, Germany

## Abstract

**Background:**

Risk adjustment and stratification play an important role in quality assurance and in clinical research. The Physiological and Operative Severity Score for the enUmeration of Mortality and morbidity (POSSUM) is a patient risk prediction model based on 12 patient characteristics and 6 characteristics of the surgery performed. However, because the POSSUM was developed for quality assessment in general surgical units, its performance within specific subgroups still requires evaluation.

The aim of the present study was to assess the accuracy of POSSUM in predicting mortality and morbidity in patients with gastric cancer undergoing D2-gastrectomy.

**Methods:**

137 patients with gastric cancer undergoing gastrectomy were included in this study. Detailed, standardized risk assessments and thorough documentation of the post-operative courses were performed prospectively, and the POSSUM scores were then calculated.

**Results:**

The 30- and 90- day mortality rates were 3.6% (n = 5) and 5.8% (n = 8), respectively. 65.7% (n = 90) of patients had normal postoperative courses without major complications, 14.6% (n = 20) had moderate and 13.9% (n = 19) had severe complications. The number of mortalities predicted by the POSSUM-Mortality Risk Score (R1) was double the actual number of mortalities occurring in the median and high-risk groups, and was more than eight times the actual number of mortalities occurring in the low-risk group (R1 < 20%). However, the calculated R1 predicted rather well in terms of severe morbidity or post-operative death in each risk group: in predicted low risk patients the actual occurrence rate (AR) of severe morbidity or post-operative death was 14%, for predicted medium risk patients the AR was 23%, and for predicted high risk patients the AR was 50% (*p *< 0.05). The POSSUM-Morbidity Risk Score (R2) overestimated the risk of morbidity.

**Conclusion:**

The POSSUM Score may be beneficial and can be used for assessment of the peri- and post-operative courses of patients with gastric carcinoma undergoing D2-gastrectomy. However, none of the scores examined here are useful for preoperative prediction of postoperative course.

## Background

Risk adjustment and stratification play an important role in quality assurance and are indispensable tools used in clinical research. The Physiological and Operative Severity Score for the enUmeration of Mortality and morbidity (POSSUM) is a patient risk prediction model based on 12 patient characteristics and 6 characteristics of the surgery performed [1]. However, because the POSSUM model was developed for quality assessment purposes in general surgical units, in order to implement it for specific subgroups of patients, its performance within such subgroups needs to be evaluated. The results of two prospective studies [2,3] showed higher mortality and morbidity rates after D2-lymphadenectomy (LAD) than after D1-lymphadenectomy for patients with gastric cancer, although there is apparently no difference in the long-term prognoses for patients after the two procedures [4,5]. However, for certain patient subgroups, radical lymph node (LN) dissection does improve prognosis. For this reason, it would be greatly beneficial to calculate the risks of morbidity and mortality for each patient preoperatively. In addition, for prognostic studies it would be useful to be able to stratify patients according to their risk factors.

In some countries, the American Society of Anesthesiologists' (ASA) classification is widely used to provide quality assurance of surgical procedures [6,7]. The ASA-score is easy to use, but the classification is not precise [8], and it does not consider the severity of surgical insult. POSSUM has been used to make comparisons between different vascular [9,10] and colorectal [11] surgical units, and to compare individual surgeons' performance within a single unit [12,13].

The aim of the present study was to assess the accuracy of the POSSUM instrument to predict mortality and morbidity in patients with gastric cancer undergoing D2-gastrectomy.

## Methods

### Patients

All patients with gastric cancer undergoing total gastrectomy (n = 123) or subtotal gastrectomy (n = 16) at the Department of Surgery, University of Cologne between January 1, 1997 and December 31, 2001 were included in this study. Preoperatively, all patients underwent esophago-gastro-duodenoscopy with biopsies and histopathologic examination. In addition, endosonography of the stomach was performed to stage the depth of tumour infiltration (T-category), and CT-scans were done to look for evidence of metastases.

### Surgical procedure and extent of lymphadenectomy

In all cases an en bloc resection of the stomach with extended D2-lymphadenectomy was performed. The lymph node dissection included compartments I and II.

Compartment I comprises all lymph node groups along the lesser curvature (No.s 1,3 and 5) and the greater curvature (No.s 2, 4, and 6) of the stomach. Compartment II comprises lymph node stations 7 to 12 according to the General Rules for the Gastric Cancer Study in Surgery and Pathology [14]. Type II (cardia) and type III (subcardial) adenocarcinomas of the gastroesophageal junction (using the Siewert/Hölscher classification system) [15] were treated with a trans-hiatal extended gastrectomy including D2-lymphadenectomy and lymph node dissection of the lower mediastinum. In the cases of subtotal gastrectomy (n = 16), only lymph node stations 3 to 6 (compartment I) and lymph node stations 7 to 12 (compartment II) were resected en bloc. Sampling of compartments III and IV nodes (No. 13 to 16) was optional. The surgeon divided the en bloc resected tissue containing lymph nodes into separate stations and assigned numbers to these stations according to the Japanese classification system [14]. Splenectomy was performed (n = 35) in cases of proximal gastric carcinoma (types II and III) and in cases of metastatic infiltration of the splenic hilar nodes (No. 10), but not as a general rule [16].

### Risk assessment

The selection of patients for surgery was based on the surgeons' "end of the bed assessment" backed by a detailed risk analysis described elsewhere [17,18]. This risk analysis has been evaluated in a prospective study. All data were routinely available. Ninety-two per cent of the operations were performed by four surgeons specializing in upper gastrointestinal surgery. Two patients were excluded from the study owing to incomplete data despite extensive tracking of case notes. The remaining 137 patients were scored retrospectively using the POSSUM-score, and the predicted risk of morbidity and death was calculated for each patient according to the following previously described logistic regression equations [1]: log e [R1/(1 - R1)] = - 7.04 + (0.13 × physiological score) + (0.16 × operative severity score) where R1 = risk of death, and log e [R2/(1 - R2)] = - 5.91 + (0.16 × physiological score) + (0.19 × operative severity score) where R2 = risk of morbidity.

Because the equations for R1 and R2 require information about the operative insult severity, and this data was not available preoperatively, we also calculated the physiological score (PPS) and in addition the V-POSSUM [19], which uses only the physiological score: log e [R3/(1 - R3)] = - 6.0386 + (0.1539 × physiological score), where R3 = risk of death.

The postoperative course was defined as (corresponds to McPeek Index 4 – 6 [20]):

1 = normal course of disease: Patient had no significant surgical or general postoperative complications.

2 = moderately favorable course of disease: Patient had postoperative complications, but the complications were treatable with appropriate therapy.

3 = poor course of disease: Patient had multiple complications that were difficult to treat with any kind of therapy.

4 = Died as a consequence of surgery (90-day mortality).

#### Definition of postoperative morbidity

Pulmonary complications: Emphysema, pneumothorax, acute pneumonia, aspiration.

Cardiac complications: Cardiovascular collapse, cardiac decompensation, bradycardia, myocardial infarction, hypertensive or hypotensive cardiovascular crisis.

Cerebral complications: Cerebral infarction, cerebral edema, organic brain syndrome.

Renal complications: Renal failure, renal bleeding, urinary tract infection.

In computing post-operative mortality, deaths occurring in and outside of the hospital were not differentiated [21]. Complications were documented using a detailed questionnaire. The severity of the post-operative course was evaluated by the treating physician while the patient was undergoing intensive care. Evaluations were based on the overall clinical impression and did not necessarily depend on the precise number of complications.

The observed and predicted operative mortality rates were compared using frequency tables. Model performance was evaluated with the Hosmer-Lemeshow 2 statistic (HL), which is a measure of calibration or goodness of fit [22]. Calibration refers to the ability of the model to assign correct outcome probabilities to patients, i.e. whether the model-estimated probability of mortality for patients with particular risk factors agrees with the actual observed mortality rate. To obtain this statistic, the estimated probability of death for each patient was computed based on the model and then stratified into different groups. The numbers of predicted and observed outcomes for each group were then evaluated statistically. Higher values of the HL statistic represent poorer model calibration.

Statistical analysis was two-sided using a significance level of 5 per cent. The Chi-Square test was calculated using the Yates correction. All calculations were performed using the computer software package SPSS © version 11 for Windows (SPSS, Chicago, Illinois, USA).

Graphical presentation of results was done with SigmaPlot Version 8.0.

## Results

The epidemiologic data of the 137 patients are shown in table 1. The mean number of resected lymph nodes was 37.7. The number of metastatic lymph nodes was an average of 7.6 (min: 0, max: 48). The median postoperative stay in the Intensive Care Unit was 1 day (min 1, max 30), and the median postoperative stay in the hospital was 17 days (min: 8 max 103).

**Table 1:** Clinico-patho logic data of 137 patients with gastric cancer and gastrectomy with D2-lym phadenectomy.

median age	65 y	range (38 – 85 y)
gender: m:f	3:2	
**pT-categ ory**	n = 137	**%**
pT1	28	20, 4
pT2	46	33, 6
pT3	52	38, 0
pT4	11	8,0

**pN-categ ory**	n = 137	**%**
pN0	47	34, 3
pN+	90	65, 7

pM0	n = 108	78, 8 %

**UICC-stage**	**n = 137**	**%**
Stage IA	20	14, 6
Stage IB	23	16, 8
Stage II	27	19, 6
Stage IIIA	15	11, 0
Stage IIIB	9	6, 6
Stage IV	43	31, 4

**Location of tumour**	**n = 137**	%
Upper third	67	48, 9
Middle third	29	21, 2
Distal third	41	29, 9

### Postoperative course

The 30-day mortality rate was 3.6% (n = 5) and the 90-day mortality rate was 5.8% (n = 8). 65.7% (n = 90) of patients had normal postoperative courses without major complications. 14.6% (n = 20) had a medium postoperative course and 13.9% (n = 19) experienced severe complications during the postoperative period. The list of surgical and systemic complications is shown in table 2.

**Table 2:** List of surgical and systemic complications in 137 patients with gastrectomy and D2-lymphadenectomy. Multiple complications are possible.

	*total*		*Postoperative Course*			
			***1***	***2***	***3***	***4***
*Surgical complications*	*n*	*%*	*n*	*n*	*n*	*n*
Relaparotomy	5	3.6	-	1	2	2
Perforation	2	1.5	-	1	-	1
Anastomic leakage	7	5.1	1	3	2	1
secondary	1	0.7	-	1	-	-
haemorrhage						
Ileus	2	1.4	-	-	1	1
Pancreatitis	5	3.6	-	-	3	2
Peritonitis	5	3.6	-	1	1	3
Wound infection	8	5.8	2	1	2	3
Abscess	3	2.2	-	1	1	1
Pancreatic fistula	13	9.4	1	8	4	-
Catheter infection	5	3.6	-	2	3	-

*Systemic complications*						
Reanimation	6	4.4	-	-	2	4
Sepsis	7	5.1	-	1	2	4
pulmonary compl.	22	16.0	2	7	9	4
cardiac compl.	23	16.8	2	4	10	7
renal compl.	5	3.6	1	1	1	2
cerebral compl.	6	4.4	1	2	2	1
Other compl.	10	7.3	6	2	2	0

### POSSUM-Score

Calculation of the POSSUM-Mortality Risk (R1) is shown in table 3. The number of mortalities predicted by the calculated R1 value was double the actual number of mortalities occurring in the median and high-risk groups, and was more than eight times the actual number of mortalities occurring in the low-risk group (R1 < 20%). The calculated R1 was much better as an estimate of severe morbidity or post-operative death, however, with predicted values matching the number of observed cases. For patients in the low-risk group (predicted risk 0 – 20%), the actual rate of severe morbidity or post-operative death was 14%; for patients in the median risk group (predicted risk 21 – 40%), the actual rate observed was 23%; and for patients in the high-risk group (predicted risk > 40%), the actual rate of severe morbidity or post-operative death was 50%. The observed rates of morbidity and mortality differed significantly between the three groups (p < 0.05).

**Table 3:** Results of POSSUM Mortality calculation (R1) compared to observed mortality (outcome 4) and to the rate of severe morbidity and death (outcome 3 +4) in 137 patients with gastrectomy and D2-lymphadenectomy.

	Possum Mortality Risk (R1)	patients	expected death	observed death ** outcome = 4**		Observed morbidity and death **outcome = 3+4**	
	(%)	n	n	n	%	n	%
	0–10	21	1	0	0	3	14,3
	38675	51	7	1	2,0	7	13,7
**low risk**	**0 – 20**	**72**		**1**	**1, 0**	**10**	**13, 9**

	21–30	38	9	3	7,8	9	23,7
	31–40	19	6	2	10,5	4	21,1
**med risk**	**21–40**	**57**		**5**	**8, 8**	**13**	**22, 8**

	41–60	4	1	1	25,0	1	25,0
	71–100	4	2	1	25,0	3	75,0
**high risk**	**> 40**	**8**		**2**	**25, 0**	**4**	**50, 0**

**total**		**137**	**29**	**8**		**27**	

The POSSUM Morbidity equation (R2) predicted nearly twice as many cases of mild or severe morbidity (including death) than were actually observed (table 4). Only for patients with a very low risk (R2 < 40%) or very high-risk (R2 > 90%), predictions were good. However, for patients with a calculated R2 less than or equal to 60% (low-risk group), 19.1% (9 of 47) actually developed complications or died after D2-resection, and for patients with higher calculated R2 values >60% (high-risk group), 42.2% (38 of 90) actually did so. The observed morbidity and mortality (for outcomes 2–4 defined above) differed significantly between these low and high-risk groups (p < 0.01). There was no significant difference measured for outcomes 3 and 4, but the test lacked sufficient power.

**Table 4:** Results of POSSUM morbidity calculation (R2) compared to morbidity rate (outcome 2, 3 and 4) of 137 patients with gastrectomy and D2-lymphadenectomy.

POSSUM Risk of Morbidity R2		Patients	Expected morbidity	observed severe morbidity and mortality **Outcome (3 + 4)**		observed morbidity and mortality **Outcome (2 – 4)**	
	%	n	n	n	%	n	%

	0 – 30	2	0	0	0	0	0
	31 – 40	12	3,9	3	25,0	4	25,0
	41 – 50	14	6,6	1	7,1	2	14,3
	50 – 60	19	10,6	2	10,5	3	15,8
**low risk**	**0 – 60**	**47**		**6**	**12, 8**	**9**	**19, 1**

	61 – 70	21	14,9	2	9,5	8	38,1
	71 – 80	35	25,7	7	20,0	14	40,0
	81 – 90	28	22,4	7	25,0	10	35,7
	91–100	6	5,7	5	83,3	6	100,0
**high risk**	**> 60**	**90**		**21**	**23, 3**	**38**	**43, 3**

	Total	137		27		47	

**Figure 1:**
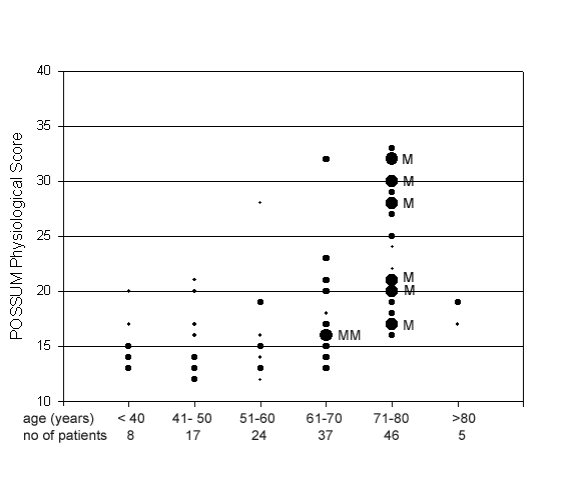
Correlation between age of the patients, POSSUM Physiological Score (PPS) and the postoperative course of 137 patients with gastrectomy and D2-lymphadenectomy: M = case with postoperative mortality, size of circles shows the number of cases with the same PPS.

However, for cases of pre-operative predictions of post-operative course using the POSSUM score, only physiologic criteria and not operative data are available for calculations. Therefore, we used the POSSUM Physiological Score (PPS) for outcome prediction. To assess the predictive value of the PPS, we used logistic regression analysis. Worse outcomes appeared to occur more frequently in patients with higher PPS scores, however this correlation was not statistically significant. The correlation between patient age, PPS-score, and mortality is shown in figure 1. There was no significant correlation between predicted risk and actual post-operative course using the calculated V-POSSUM score (data is not shown).

## Discussion

For patients with gastric cancer, especially in advanced stages, extensive lymphadenectomy (LAD) can improve prognosis. The results of the Dutch prospective randomized study show comparable long-term outcomes for patients undergoing either D1- or D2-lymphadenectomy [23]. However, this study also demonstrated an increased rate of morbidity and mortality for patients undergoing D2-LAD versus D1-LAD [4]. Therefore, it is important to know pre-operatively which patients are more likely to benefit from the more radical operation. Furthermore, in order to compare study results for outcomes, it is necessary to stratify the investigated patients according to risk profiles.

Patients with gastric carcinoma are usually older than 60 years of age and have corresponding concomitant medical problems, which may significantly influence the post-operative course. For example in our study, 6 of the 8 post-operative mortalities occurred in patients between 70 and 80 years of age. The risks for such older patients could be only partially evaluated using the POSSUM Physiologic Score (PPS). The lack of significant correlation between higher PPS scores and higher risk for older patients may be due to the small number of participants in this study.

There are various established assessment systems designed to assess the gravity of pre-existing illnesses. The ASA-Classification Score, for example, is used most often for this purpose in surgical and anesthesia settings [6,7]. This score was developed by anesthesiologists to consider the risks of anesthetic procedure. It functions well as a fast assessment of patients, and should ascertain whether a life-threatening condition exists or whether peri-operative problems can be expected. However, the ASA-score is less suitable to determine whether a patient will develop serious complications as a result of the magnitude of the operation performed [8]. In our study, half of the patients were classified as ASA II, and half as ASA III. This risk stratification correlated only weakly with the patients' actual post-operative course.

The POSSUM Score has been evaluated in numerous studies [1,10-13,24]. The main objective in these studies was to ascertain whether this score is suitable to evaluate the *Case-Mix *[1,10,13,19]. It has also been used to assess surgeon-dependent risk factors [12,13]. A number of studies have evaluated the applicability of the POSSUM Score for particular medical conditions, and therefore a number of varieties have emerged, i.e. the P-Possum-Score [25] or the V-POSSUM-Score [19]. As shown in the foregoing results, the POSSUM Mortality Index (R1) calculated the probability of mortality for patients with gastric carcinoma undergoing D2-LAD at two to three times the actual rates occurring in our patient population. Although this index was developed to predict the 30-day mortality rate, we applied it to the 90-day mortality rate as well. Because of the low mortality rate in our patient cohort, the difference (3.6% – 5.1%) was irrelevant. An overestimate of mortality risk using this index has also been found in other studies, i.e. for patients with esophageal carcinoma, where a high post-operative mortality is expected [26,27].

Despite these discouraging results, there was a significant correlation of the predicted risk of severe morbidity (including mortality) with the actual incidence using the R1 index. This correlation was evident in all three of the classified risk levels, where the observed outcomes agreed completely with the predicted risk for severe morbidity. In light of these results, the R1 classification appears to be suitable for risk stratification purposes in clinical studies.

The calculated POSSUM-Morbidity Index (R2) overestimated the risk of developing post-operative complications in this study. There is an acceptable correlation between predicted values and observed rates of morbidity when using the R2 to evaluate both very low risk patients and very high-risk patients. In the other categories, however, the risk was overestimated two to three-fold.

The POSSUM Score is used to predict the post-operative course of patients, using both the pre-operative assessment of the severity of pre-existing concomitant medical conditions as well as information gathered during the peri-operative period i.e. severity of surgical insult, intra-operative blood loss, etc. However, peri-operative information was not available pre-operatively; the decision regarding magnitude of gastrectomy had not yet been made. Therefore, our investigation focused on whether application of the POSSUM Physiological Score would be sufficient to make an accurate pre-operative prediction. The V-POSSUM Score, which was previously evaluated primarily for the assessment of patients with vascular disease [24], was ineffective for predicting post-operative course in our patient cohort. Using this score we showed, admittedly, that patients with high PPS scores also had higher risks of complications or post-operative mortality, but the correlation was not significant enough to give a valid pre-operative risk assessment.

There are still more instruments that may be used for risk assessment of patients with concomitant medical problems. For example, the Charlson Comorbidity Index (CCI) was developed particularly to address this issue [28,29]. This assessment focuses primarily on long-term outcomes. However, some studies have shown that the CCI is not suitable for the prediction of post-operative course [8]. Another assessment instrument, the APACHE II Score, was developed for patients in the Intensive Care Unit to predict the courses of patients there [30,31]. Unfortunately, the APACHE Score is irrelevant in our patient cohort, where some of the assessed parameters do not exist pre-operatively, or change post-operatively.

## Conclusion

This study shows that the POSSUM Mortality Score (R1) is a suitable instrument to risk stratify patients with gastric carcinoma undergoing D2-LAD for the development of severe post-operative complications (including post-operative mortality), based on pre-existing or concomitant medical problems. The POSSUM Morbidity Index (R2) is particularly suitable for risk assessment if the target parameters include moderate to severe complications. When using this instrument, however, the overestimation of risk must be considered. For our purposes, none of the instruments (i.e. PPS, V-POSSUM) examined for pre-operative risk assessment were effective models. Finally, in our study we showed that accurate documentation of standardized risk scores is possible under routine conditions and that the necessary parameters for a second score like the POSSUM Score could be formulated.

## Competing interests

The author(s) declare that they have no competing interests.

## Authors' contributions

EB conceived the study, performed the statistical analysis and drafted the manuscript.

TL documented the risk factors and complications

SM participated in the design and coordination of the study

AH participated in the design of the study and carried out surgery

All authors read and approved the final manuscript.

## Pre-publication history

The pre-publication history for this paper can be accessed here:


